# Rapidly expanding spin-polarized exciton halo in a two-dimensional halide perovskite at room temperature

**DOI:** 10.1126/sciadv.abp8135

**Published:** 2022-07-29

**Authors:** Go Yumoto, Fumiya Sekiguchi, Ruito Hashimoto, Tomoya Nakamura, Atsushi Wakamiya, Yoshihiko Kanemitsu

**Affiliations:** Institute for Chemical Research, Kyoto University, Uji, Kyoto 611-0011, Japan.

## Abstract

Monitoring of the spatially resolved exciton spin dynamics in two-dimensional semiconductors has revealed the formation of a spatial pattern and long-range transport of the spin-polarized excitons, which holds promise for exciton-based spin-optoelectronic applications. However, the spatial evolution has been restricted to cryogenic temperatures because of the short exciton spin relaxation times at room temperature. Here, we report that two-dimensional halide perovskites can overcome this limitation owing to their relatively long exciton spin relaxation times and substantial exciton-exciton interactions. We demonstrate the emergence of a halo-like spatial profile in spin-polarized exciton population and its ultrafast expansion at room temperature by performing time-resolved Faraday rotation imaging of spin-polarized excitons in two-dimensional perovskite (C_4_H_9_NH_3_)_2_(CH_3_NH_3_)_3_Pb_4_I_13_. Exciton-exciton exchange interactions induce density-dependent nonlinear relaxation and ultrafast transport of exciton spins and give rise to a rapidly expanding halo-like spatial pattern. The density-dependent spatial control suggests the potential of using two-dimensional halide perovskites for spin-optoelectronic applications.

## Teaser

2D halide perovskites realize room temperature spatial evolution of exciton spins induced by exciton-exciton interactions.

## INTRODUCTION

Observation of spatial dynamics of spin-polarized carriers provides crucial information for understanding spin physics in semiconductors and developing spintronic devices. Spatially resolved optical measurements have clarified a number of fascinating spin-related phenomena (*1*–*8*). Spin-polarized excitons in atomically thin two-dimensional (2D) semiconductors have attracted considerable interest for spin-optoelectronic applications owing to their large binding energy and unique spin-dependent characters (*9*, *10*). Monitoring of the spatial dynamics of the spin-polarized excitons in 2D transition metal dichalcogenides (TMDCs) has shown the formation of spatial patterns (*5*, *6*) and long-range exciton spin transport (*8*). These findings elucidated the exciton spin transport mechanisms and suggested the potential of using 2D semiconductors for exciton-based spin-optoelectronic applications. However, observations of such spatial dynamics have been restricted to cryogenic temperatures (*5*, *6*, *8*) because the short exciton spin relaxation time at room temperature prevents the formation of a spatial pattern and transport of the spin-polarized excitons (*6*, *8*). The absence of spatial evolution of spin-polarized excitons at room temperature limits the potential for applications such as information and signal processing (*8*) and chiral nanophotonics (*11*).

Exciton-exciton interactions can induce the fast expansion of spin-polarized excitons (*3*, *5*, *8*, *12*) and thus are expected to open the route for accessing their spatial degree of freedom at room temperature. 2D Ruddlesden-Popper lead halide perovskites (2D RPPs), described by the formula L_2_A_*n*−1_Pb*_n_*X_3*n*+1_ (L is a long organic cation, A is a cation, X is a halide, and *n* is an integer), have attracted growing attention due to their excellent optical (*13*) and optoelectronic (*14*, *15*) properties. Recently, 2D RPPs have also been recognized as fascinating spin-optoelectronic materials with spin-dependent optical transitions (*13*, *16*–*24*). Alternate stacking of 2D perovskite and long organic barrier layers constitutes the self-assembled multiple quantum well structure (*25*), and the electrons and holes are confined within a 2D perovskite layer A_*n*−1_Pb*_n_*X_3*n*+1_, which is composed of *n* layers of corner-sharing [PbX_6_]^4−^ octahedra. Because of the quantum and dielectric confinement effects (*26*–*29*), stable excitons with large binding energies form even for 2D RPPs with *n* > 1 (*29*) and dominate the optical and optoelectronic properties at room temperature (*21*, *23*, *26*, *28*–*32*). It has recently been reported that exciton-exciton interactions significantly influence the dynamics of the excitons and their spins (*21*, *23*, *30*–*32*). In addition to the substantial effect of exciton-exciton interactions, an increase in the exciton spin relaxation time has been reported in 2D RPPs with larger *n* (*18*). These properties make 2D RPPs promising candidates for demonstrating exciton spin transport at room temperature and enhancing the potential of using exciton spins as information carriers in spin-optoelectronic devices. Therefore, it is of great importance to reveal spatial and temporal dynamics of spin-polarized excitons in 2D RPPs.

In the work reported here, we performed time-resolved Faraday rotation imaging of spin-polarized excitons in 2D RPPs at room temperature and demonstrated the formation of a halo-like spatial profile in the spin-polarized exciton population and its ultrafast expansion. We found that the rapidly expanding halo-like spatial profile originates from density-dependent nonlinear relaxation and ultrafast transport of the spin-polarized excitons, both of which are induced by exciton-exciton exchange interactions. These findings reveal the impact of the exciton-exciton interactions on the spatiotemporal dynamics of exciton spins at room temperature. The density-dependent spatial control of the exciton spins suggests the potential of using 2D RPPs for spin-optoelectronic applications.

## RESULTS

### Experimental setup and sample characterizations

The band-edge states in 2D RPPs consist of *s*-like valence band states arising from Pb 6s and I 5p orbitals and split-off conduction band states that are split from the Pb 6p conduction band by the strong spin-orbit coupling (*25*, *33*, *34*). Because both the band-edge valence and conduction band states have a total angular momentum of ^1^/_2_, the bright band-edge excitons are characterized by a total exciton angular momentum projection *M*_ex_ of +1 or −1, which can be excited by right-handed (σ^+^) or left-handed (σ^−^) circularly polarized light, respectively (*13*, *17*, *18*, *20*, *21*, *23*, *35*–*37*). Therefore, the difference between the exciton spin populations with *M*_ex_ = +1 (*N*_+_) and *M*_ex_ = −1 (*N*_−_) represents the spin-polarized exciton population *N*_+_ − *N*_−_. Because the Faraday rotation is proportional to *N*_+_ − *N*_−_ (*35*, *36*), we performed time-resolved imaging of the pump-induced Faraday rotation angle θ_F_ and investigated the spatiotemporal dynamics of the spin-polarized excitons.

**Fig. 1.**
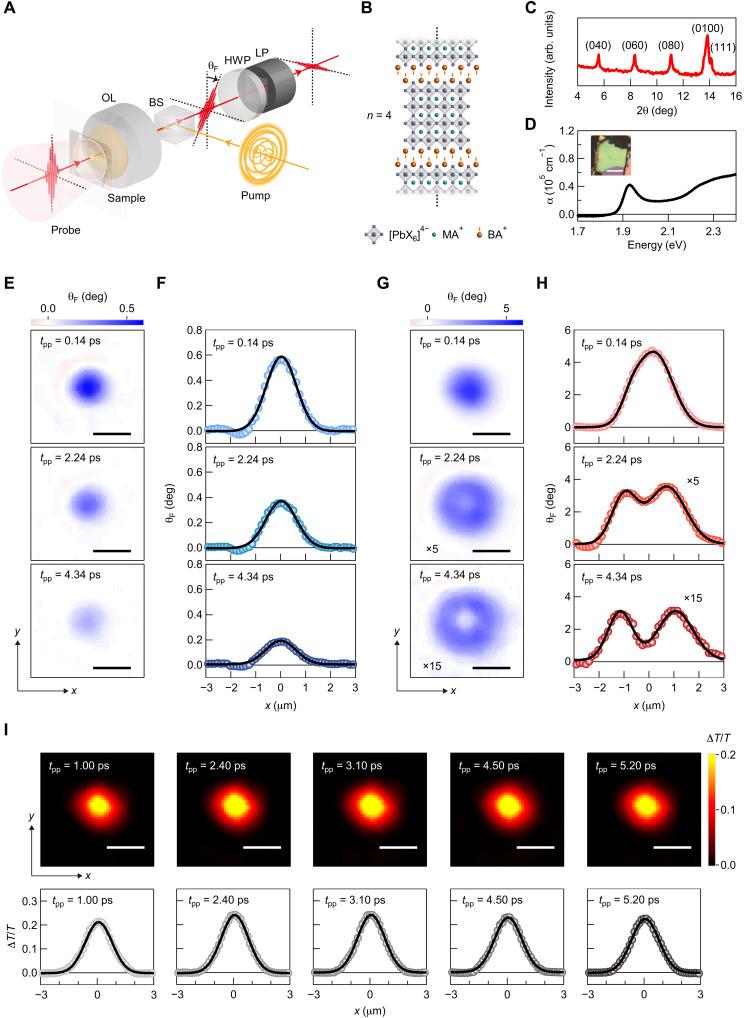
Time-resolved Faraday rotation imaging of spin-polarized excitons in 2D RPP (BA)_2_(MA)_3_Pb_4_I_13_. (**A**) Illustration of the time-resolved Faraday rotation imaging setup. OL, objective lens; BS, beam splitter; HWP, half-wave plate; LP, linear polarizer. (**B**) Schematic crystal structure of 2D RPP (BA)_2_(MA)_3_Pb_4_I_13_ (*n* = 4). (**C**) X-ray diffraction spectrum of (BA)_2_(MA)_3_Pb_4_I_13_ crystals. The vertical axis is in log scale. (**D**) Absorption coefficient spectrum of an exfoliated sample. The inset is an optical microscope image of the sample. Scale bar, 10 μm. (**E**) Images of pump-induced Faraday rotation angle at pump-probe delay times of 0.14, 2.24, and 4.34 ps for an exciton density per 2D perovskite layer of 6.8 × 10^11^ cm^−2^. Scale bars, 2 μm. (**F**) Spatial profiles of pump-induced Faraday rotation angle, which correspond to the horizontal cross sections of the images in (E) passing through the center of the excitation spot (*x* = *y* = 0 μm). The black curves are Gaussian fits. (**G** and **H**) Same as (E) and (F) but for an exciton density per 2D perovskite layer of 1.2 × 10^13^ cm^−2^. The middle and bottom panels of (G) and (H) are magnified by factors of 5 and 15, respectively. The black curves in (H) are double-Gaussian fits. (**I**) Top: Time-resolved images of pump-induced change in transmission at various pump-probe delay times for *n*_ex_ = 1.3 × 10^13^ cm^−2^. Scale bars, 2 μm. Bottom: Spatial profiles of pump-induced change in transmission, which correspond to the horizontal cross sections of the images shown in the top panels passing through the center of the excitation spot (*x* = *y* = 0 μm). The black curves are Gaussian fits.

To achieve millidegree, submicrometer, and subpicosecond resolutions within a short measurement time, we used a direct imaging technique with the rotation analyzer method (see Fig. 1A and Materials and Methods) (*38*). The spin-polarized excitons were generated by using an objective lens to focus circularly polarized pump pulses onto the sample. The pump-induced spin-polarized exciton population was detected by using linearly polarized probe pulses, which counter-propagated with the pump pulses and were loosely focused onto the sample. The spot sizes of the pump and probe beams (full width at half maximum) were 1.1 and 40 μm, respectively. The spatial profile of θ_F_ was measured by using a complementary metal-oxide semiconductor (CMOS) camera to image the probe pulses after they had passed through an analyzer composed of an achromatic half-wave plate (HWP) and a linear polarizer (LP).

We synthesized phase-pure 2D RPP single crystals with *n* = 4 (BA)_2_(MA)_3_Pb_4_I_13_ (see Fig. 1B and Materials and Methods), where BA and MA denote butylammonium (C_4_H_9_NH_3_) and methylammonium (CH_3_NH_3_) cations, respectively. It has been reported that 2D RPPs with larger *n* exhibit higher environmental stability (*39*, *40*). We observed that (BA)_2_(MA)_*n*−1_Pb*_n_*I_3*n*+1_ crystals with smaller *n* were more easily damaged during our measurements. In addition, the synthesis of phase-pure 2D RPPs with *n* ≥ 5 requires more precise stoichiometric or chemical control (*25*). Therefore, we chose (BA)_2_(MA)_3_Pb_4_I_13_ for our experiments. Figure 1C shows the x-ray diffraction spectrum of the sample, which ensures its *n* = 4 phase purity (*25*, *29*, *32*). We mechanically exfoliated the (BA)_2_(MA)_3_Pb_4_I_13_ crystal flakes and transferred them onto a glass substrate. To obtain higher environmental stability and exclude extrinsic effects such as surface relaxation (*41*), we investigated crystal flakes much thicker than monolayers. Figure 1D shows the absorption coefficient spectrum of an exfoliated flake (see Materials and Methods), whose thickness was estimated to be 150 nm from atomic force microscopy measurements (see fig. S1). The absorption peak at *E*_0_ = 1.93 eV corresponds to the 1s exciton energy of (BA)_2_(MA)_3_Pb_4_I_13_ (*29*), and the other absorption peaks for (BA)_2_(MA)_*n* − 1_Pb*_n_*I_3*n* + 1_ with *n* ≠ 4 cannot be discerned, which further confirms the *n* = 4 phase purity.

### Time-resolved Faraday rotation imaging of spin-polarized excitons

In the time-resolved imaging experiments, we fixed the polarization of the pump pulses to σ^+^ circular polarization because θ_F_ does not depend on the pump helicity, except for the sign of θ_F_ (see text S1). The pump energy was set to be 1.93 eV because the resonant excitation efficiently generates the spin-polarized excitons (*21*, *42*). We set the probe energy to be 1.83 eV, which is below *E*_0_, to filter out only the pump pulses for the detection of the probe pulses and avoid the generation of the excitons by the probe pulses. The measured θ_F_ does not depend on the intensity of the probe pulses in our measurements (see text S2). Figure 1 (E and G) illustrates the time-resolved images of θ_F_ obtained with the flake shown in Fig. 1D at room temperature for low and high excitation intensities, which correspond to exciton densities per 2D perovskite layer *n*_ex_ of 6.8 × 10^11^ and 1.2 × 10^13^ cm^−2^, respectively (the procedure for estimating *n*_ex_ is provided in text S3). The free carrier fractions for *n*_ex_ = 6.8 × 10^11^ and 1.2 × 10^13^ cm^−2^ are estimated from the 2D Saha equation (*43*) to be 0.06 and 0.01, respectively. Here, we used the exciton binding energy and reduced mass of 157 meV and 0.196*m*_0_ (*m*_0_ is the free electron mass) (*29*), respectively, and the temperature of 300 K. These fractions show that the contribution of the free carrier is negligible in our observations. In addition, the exciton Bohr radius *a*_B_ in (BA)_2_(MA)_3_Pb_4_I_13_ is ~1 nm (*29*), which leads to the Mott density of around *a*_B_^−2^ = 1 × 10^14^ cm^−2^ (*32*). All the measurements were performed for *n*_ex_ more than one order below the Mott density. Figure 1E shows that for *n*_ex_ = 6.8 × 10^11^ cm^−2^, the spin-polarized exciton population decays, keeping its isotropic Gaussian spatial profile around the center of the excitation spot (*x* = *y* = 0 μm). This is shown by the slices of the spatial profiles at *y* = 0 μm, which are well reproduced by a Gaussian function (Fig. 1F). For *n*_ex_ = 1.2 × 10^13^ cm^−2^ (Fig. 1G), we observed a single-peak spatial profile at a pump-probe delay time *t*_pp_ of 0.14 ps and found that the peak θ_F_ takes a large value of 4.71 deg, corresponding to 31 deg μm^−1^. As *t*_pp_ is increased, in stark contrast to the case of *n*_ex_ = 6.8 × 10^11^ cm^−2^, we found that the spatial profile evolves into a halo-like spatial pattern. The slices at *y* = 0 μm show a double-peak structure and are well reproduced by a double-Gaussian function (Fig. 1H). Here, we note that the observed halo-like spatial pattern does not originate from sample degradation during the measurements. In addition, we observed that the halo formation also appears in different exfoliated flakes, and *n*_ex_ for the emergence of the halo-like spatial profile does not vary between different flakes (see text S4). Therefore, we found that at high exciton density, a halo-like spatial pattern emerges in the spin-polarized exciton population within a picosecond time scale at room temperature.

To study the total exciton population (*N*_+_ + *N*_−_) when the halo-like spatial pattern appears in the spin-polarized exciton population, we performed time-resolved imaging of pump-induced change in transmission Δ*T*/*T* on the flake shown in Fig. 1D (see text S5 for details). In Fig. 1I, we display the time-resolved images of Δ*T*/*T* at different *t*_pp_ for *n*_ex_ = 1.3 × 10^13^ cm^−2^. Because Δ*T*/*T* reflects *N*_+_ + *N*_−_, we can see that even for *n*_ex_ = 1.3 × 10^13^ cm^−2^, a halo-like spatial pattern does not appear in the total exciton population, which is in stark contrast to the case of the spin-polarized exciton population. We note that the halo-like spatial pattern of the total exciton population has been reported in a TMDC monolayer (*44*), and its origin has been explained by the phonon-mediated processes such as phonon wind and drag (*45*) and thermal drift (*46*), which drive the excitons away from the excitation spot. However, in our experiments, the halo formation is observed only in the spin-polarized exciton population, and it cannot be identified in the total exciton population. Therefore, the phonon-induced effects can be considered to be negligible. In addition, we observed a Gaussian-shaped spatial profile and negligible decay of Δ*T*/*T* within the exciton spin relaxation time (see Fig. 1I and text S5). Although it is known that Auger recombination causes density-dependent exciton recombination (*31*, *32*, *44*), the negligible decay of Δ*T*/*T* after the pump excitation shows that the Auger recombination has little effect on our observations.

**Fig. 2.**
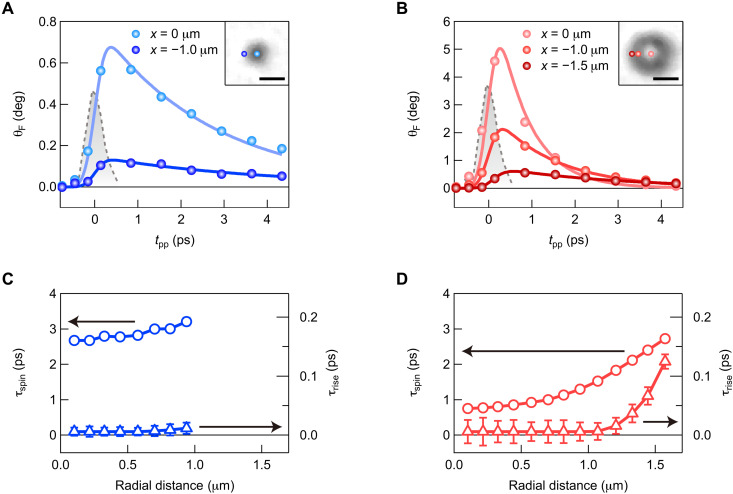
Spatially dependent dynamics of spin-polarized exciton population. (**A**) Pump-induced Faraday rotation angle as a function of pump-probe delay time, obtained at positions of *x* = 0 and −1.0 μm with *y* = 0 μm for an exciton density per 2D perovskite layer of 6.8 × 10^11^ cm^−2^. The solid curves are fits to the data with an exponential rise and decay function convoluted with a Gaussian response function. The gray shaded area represents the cross-correlation between the pump and probe pulses. The inset shows the corresponding positions in an image of pump-induced Faraday rotation angle at a pump-probe delay time of 4.34 ps. Scale bar, 2 μm. (**B**) Same as (A) but obtained at positions of *x* = 0, −1.0, and − 1.5 μm with *y* = 0 μm for an exciton density per 2D perovskite layer of 1.2 × 10^13^ cm^−2^. (**C**) Estimated exciton spin relaxation time (open circles; left axis) and rise time (open triangles; right axis) as a function of radial distance from the center of the excitation spot for an exciton density per 2D perovskite layer of 6.8 × 10^11^ cm^−2^. Error bars are the SD of the fitting parameters. Some error bars are smaller than the symbols. (**D**) Same as (C) but for an exciton density per 2D perovskite layer of 1.2 × 10^13^ cm^−2^.

To investigate the spatiotemporal dynamics of the spin-polarized excitons, we plotted the *t*_pp_ dependence of θ_F_ at different spatial positions for *n*_ex_ = 6.8 × 10^11^ and 1.2 × 10^13^ cm^−2^ in Fig. 2 (A and B), respectively. As can be seen from the solid curves in Fig. 2 (A and B), all the temporal dynamics of θ_F_ can be well fitted with a convolution between a Gaussian response function and an exponential rise and decay function, exp( − *t*/τ_spin_) × [1 − exp(− *t*/τ_rise_)]. Here, τ_spin_ is the decay time corresponding to the exciton spin relaxation time and τ_rise_ is the rise time. We determined the Gaussian response function (gray shaded areas in Fig. 2, A and B) from the cross-correlation between the pump and probe pulses and estimated its full width at half maximum to be 460 fs (see text S6).

To study the spatial dependences of τ_spin_ and τ_rise_, we performed the fitting over the whole imaging area. For better signal-to-noise ratio, we discuss τ_spin_ and τ_rise_ as a function of radial distance *r*, which were obtained by averaging the fitting results at the same radial distance from the center of the excitation spot. The estimated *r* dependences of τ_spin_ and τ_rise_ for *n*_ex_ = 6.8 × 10^11^ cm^−2^ are plotted in Fig. 2C. While τ_spin_ shows a slight *r* dependence around 3 ps, τ_rise_ takes small values over the entire range of *r* and is zero within the error bars at *r* < 0.9 μm. The zero τ_rise_ means that the spin-polarized excitons are instantaneously generated within the cross-correlation time between the pump and probe pulses. On the other hand, as shown in Fig. 2D, both τ_spin_ and τ_rise_ show substantial *r* dependences for *n*_ex_ = 1.2 × 10^13^ cm^−2^. It can be seen that τ_spin_ is largely suppressed near the center of the excitation spot and with increasing *r* approaches its value for *n*_ex_ = 6.8 × 10^11^ cm^−2^. In τ_rise_, a notable deviation from zero appears at *r* > 1.2 μm, meaning that the generation of the spin-polarized exciton population is not completed even after the pump excitation. The finite τ_rise_ outside the excitation spot shows the occurrence of ultrafast exciton spin transport, which is further discussed below. We note that, although the ultrafast exciton spin transport would decrease the spin-polarized exciton population inside the excitation spot, the temporal decay of θ_F_ at every spatial position is well reproduced by a single decay component of τ_spin_. The absence of the fast decay component originating from the ultrafast exciton spin transport shows that inside the excitation spot, the amount of change in the spin-polarized exciton population induced by the ultrafast transport is negligible compared to that induced by the exciton spin relaxation. Therefore, the estimated τ_spin_ is considered to directly reflect the exciton spin relaxation time.

### Density-dependent nonlinear exciton spin relaxation

**Fig. 3.**
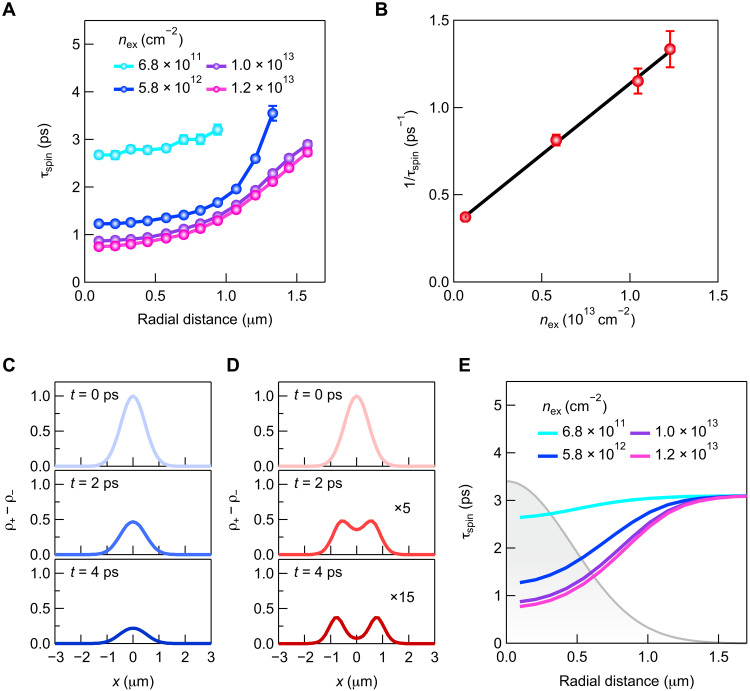
Density-dependent exciton spin relaxation and formation of spin-polarized exciton halo. (**A**) Radial distance dependence of exciton spin relaxation time for different exciton densities. Error bars are the SD of the fitting parameter. Some error bars are smaller than the symbols. (**B**) Exciton density dependence of estimated exciton spin relaxation rate at a radial distance of 0.1 μm. The black line is a linear fit. Error bars are estimated from those in (A). (**C**) Calculated time evolution of spatial profiles of normalized spin-polarized exciton density at *y* = 0 μm for an exciton density per 2D perovskite layer of 6.8 × 10^11^ cm^−2^. (**D**) Same as (C) but for an exciton density per 2D perovskite layer of 1.2 × 10^13^ cm^−2^. The middle and bottom panels are magnified by factors of 5 and 15, respectively. (**E**) Calculated radial distance dependence of exciton spin relaxation time for different exciton densities. The gray shaded area depicts the pump beam profile.

Because of the substantial dependences of τ_spin_ on *r* and *n*_ex_, the nonlinear exciton spin relaxation is considered to be the origin of the observed halo-like spatial pattern. To further study the nonlinear exciton spin relaxation, we measured the *r* dependence of τ_spin_ for different *n*_ex_ (Fig. 3A). We observed that with increasing *n*_ex_, τ_spin_ decreases and the *r* dependence becomes significant. Figure 3B shows the *n*_ex_ dependence of the exciton spin relaxation rate 1/τ_spin_ at the center of the excitation spot (*r* = 0.1 μm), and we found a linear dependence of 1/τ_spin_ on *n*_ex_. Fitting to the data in Fig. 3B with a linear function of (1/τ_spin_^0^) + β*n*_ex_, we obtained a τ_spin_ at the low exciton density limit, τ_spin_^0^, of 3.1 ps and a proportionality constant β of 8.14 × 10^−2^ cm^2^ s^−1^. The observed linear dependence is assigned to the bimolecular exciton spin relaxation process induced by exciton-exciton exchange interaction (*21*, *23*, *42*, *47*, *48*), which originates from Coulomb interactions between electrons and holes forming two excitons. This *n*_ex_-dependent spin relaxation has been reported to occur in GaAs quantum wells (*47*), TMDCs (*48*), and 2D lead halide perovskites (*21*, *23*, *42*). The estimated value of β = 8.14 × 10^−2^ cm^2^ s^−1^ is one order of magnitude larger than the reported exciton-exciton annihilation (Auger recombination) rate of 2.8 × 10^−3^ cm^2^ s^−1^ (*32*), which supports the fact that Auger recombination has little effect on the nonlinear exciton spin relaxation.

The proposed mechanism of the formation of the halo-like spatial pattern was confirmed by performing numerical simulations using 2D diffusion equations for the exciton densities with *M*_ex_ = ±1 (*n*_±_)

$$\frac{\partial n_{\pm }}{\partial t}=D\nabla^{2}n_{\pm }-\frac{n_{\pm }}{\tau }\mp \frac{\gamma_{s}}{2}(n_{+}-n_{-})$$ (1)

where *D* is the diffusion constant, τ is the exciton recombination time, and γ_s_ is a coefficient representing the *n*_ex_-dependent exciton spin relaxation. γ_s_ is given by (see Materials and Methods)

$$\gamma_{s}=\frac{1}{\tau_{\text{spin}}^{0}}+2\beta (n_{+}+n_{-})$$ (2)

Assuming that the initial exciton densities are given by $n_{+}(t=0,x,y)=\frac{n_{\text{ex}}}{2}\times \text{exp}(-\frac{x^{2}}{2{\sigma_{x,0}}^{2}}-\frac{y^{2}}{2{\sigma_{y,0}}^{2}})$ and *n*_−_(*t* = 0, *x*, *y*) = 0, we calculated the time evolution of the spatial profile of the spin-polarized exciton population. The factor of ^1^/_2_ in the amplitude of *n*_+_(*t* = 0, *x*, *y*) was introduced, so that the spatial integral of *n*_+_(*t* = 0, *x*, *y*) is equal to the total exciton population *n*_ex_πσ_*x*,0_σ_*y*,0_ (see text S3). In the calculations, we used σ_*x*,0_ = 0.46 μm and σ_*y*,0_ = 0.44 μm, corresponding to the excitation spot size. The spot size was measured by imaging the pump beam reflected from the sample. We set *D* and τ to the literature values of 0.25 cm^2^ s^−1^ and 5 ns, respectively (*32*). These values indicate the negligible contributions of the first and second terms on the right-hand side of Eq. 1 within the picosecond time scale, which can be seen from the observed spatiotemporal dynamics of the total exciton population (see text S5). Figure 3 (C and D) shows the calculated spatial profiles of normalized spin-polarized exciton density ρ_+_ − ρ_−_ at *y* = 0 μm for *n*_ex_ = 6.8 × 10^11^ and 1.2 × 10^13^ cm^−2^, respectively. Here, we defined the normalized *n*_±_ as ρ_±_ = 2*n*_±_/*n*_ex_. We found that the calculated results reproduce the observed spatial behavior shown in Fig. 1 (F and H) and the formation of the halo-like spatial pattern. In Fig. 3E, we plot the simulated *r* dependence of τ_spin_ for different *n*_ex_, which reflects the pump beam profile and reproduces the overall behavior of the experimental data shown in Fig. 3A. These results clarify the dominant contribution of the density-dependent exciton spin relaxation to the halo formation.

### Ultrafast exciton spin transport

**Fig. 4.**
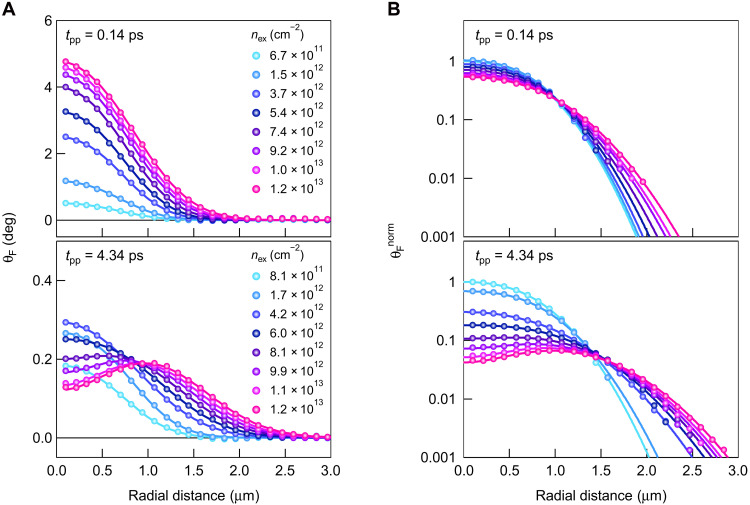
Exciton density dependence of spatial profiles of pump-induced Faraday rotation and ultrafast exciton spin transport. (**A**) Radial profiles of pump-induced Faraday rotation angle for different exciton densities at pump-probe delay times of 0.14 ps (top) and 4.34 ps (bottom). The curves are double-Gaussian fits. (**B**) Radial profiles of pump-induced Faraday rotation angle obtained by normalizing the spatial profiles in (A) by each exciton density. At each pump-probe delay time, all normalized spatial profiles are further multiplied by a factor that makes the peak value for the lowest exciton density equal to 1. The normalized pump-induced Faraday rotation angle is shown in log scale. The curves are double-Gaussian fits.

Despite the good agreement between the experiments and calculations, the spatial extent of the spin-polarized exciton population is underestimated by the calculations, especially for higher exciton density (see text S7). The broader spatial profiles observed in the experiments are considered to reflect the fact that ultrafast exciton spin transport occurs and becomes more prominent for higher *n*_ex_. To elucidate the *n*_ex_-dependent expansion of the spin-polarized exciton population, we measured the *r* dependences of θ_F_ for various *n*_ex_ at *t*_pp_ = 0.14 and 4.34 ps (Fig. 4A), which were obtained by azimuthally averaging the images of θ_F_. As shown by the curves in Fig. 4A, the radial profiles are well reproduced by a double-Gaussian function. At *t*_pp_ = 0.14 ps, θ_F_ increases with *n*_ex_, keeping the radial profiles peaked at *r* = 0.1 μm. By contrast, at *t*_pp_ = 4.34 ps, a nonmonotonic *n*_ex_ dependence of θ_F_ is observed around the center of the excitation spot while θ_F_ monotonically increases with *n*_ex_ at *r* > ~1 μm. This indicates that the dominant process determining the dynamics of the spin-polarized exciton population differs near and outside the excitation spot.

The spatially different dynamics are clarified by the radial profiles of *n*_ex_-normalized θ_F_, θ_F_^norm^ (Fig. 4B). With increasing *n*_ex_, the decrease and increase in θ_F_^norm^ are observed at shorter and longer *r*, respectively, and this trend is more pronounced at *t*_pp_ = 4.34 ps than at *t*_pp_ = 0.14 ps. If the density-dependent processes, such as the nonlinear exciton spin relaxation and the exciton spin transport, do not occur, then θ_F_ is proportional to the initially generated spin-polarized exciton population, which linearly depends on *n*_ex_. In that case, the radial profiles of θ_F_^norm^ would not depend on *n*_ex_. Therefore, the observed *n*_ex_ dependence shows the contributions of the density-dependent processes. The decrease in θ_F_^norm^ results from the faster exciton spin relaxation due to the exciton-exciton exchange interaction with increasing *n*_ex_. On the other hand, the increase in θ_F_^norm^ cannot be attributed to such a nonlinear exciton spin relaxation but can be explained by the ultrafast exciton spin transport. Therefore, the spin-polarized exciton population propagates longer with increasing *n*_ex_ and reaches around *r* = 3 μm for *n*_ex_ = 1.2 × 10^13^ cm^−2^.

## DISCUSSION

Here, we discuss possible mechanisms of the *n*_ex_-dependent ultrafast exciton spin transport: ballistic transport of nonequilibrium carriers (*49*–*51*) and spin-polarized exciton current driven by a repulsive exciton-exciton exchange interaction (*5*). It has been reported that ballistic transport of photogenerated nonequilibrium carriers (not excitons) occurs in 3D lead halide perovskites (*49*–*51*) and the ballistic transport length decreases with increasing carrier density due to enhanced carrier-carrier scattering (*50*). Ballistic transport may expand the spatial profile of the spin-polarized exciton population. However, because of the opposite dependence on the carrier density, it cannot be the origin of the observed *n*_ex_ dependence of the ultrafast exciton spin transport. On the other hand, a repulsive exciton-exciton exchange interaction gives rise to a rapid expansion of the spin-polarized exciton population with increasing *n*_ex_, where the gradient of *n*_+_ (*n*_−_) drives the repulsive force between the excitons with *M*_ex_ = +1 (−1) (*5*, *52*). Therefore, the observed *n*_ex_-dependent ultrafast exciton spin transport can be considered to originate from the repulsive exciton-exciton exchange interaction, which enables efficient transport of exciton spins at higher exciton density. Along with the ultrafast exciton spin transport, the expansion of the total exciton population is also expected because both of the exciton populations with *M*_ex_ = +1 and −1 propagate by the repulsive force. We observed the *n*_ex_-dependent ultrafast transport of the total exciton population within the time window corresponding to τ_spin_ by measuring the radial profiles of *n*_ex_-normalized Δ*T*/*T* (see text S8). This agrees with the interpretation that the ultrafast exciton spin transport is driven by the repulsive exciton-exciton exchange interaction.

The repulsive exciton-exciton exchange interaction can induce a halo-like spatial pattern in the exciton spin polarization (*n*_+_ − *n*_−_)/(*n*_+_ + *n*_−_) (*5*). However, because the spin-polarized exciton current driven by the gradient of *n*_±_ smooths the spatial profile of *n*_±_, the repulsive interaction does not cause the halo-like spatial pattern in the spin-polarized exciton population. In this study, we found that the density-dependent exciton spin relaxation results in the halo-like spatial pattern of the spin-polarized exciton population while the repulsive interaction drives the ultrafast exciton spin transport. Cooperation of the effects originating from exciton-exciton exchange interactions is the cause of the rapidly expanding spin-polarized exciton halo at room temperature. Along with the observed large Faraday rotation, this finding reveals the unique room temperature exciton spin properties in 2D RPPs.

## MATERIALS AND METHODS

### Time-resolved imaging of pump-induced Faraday rotation

We used a Yb:KGW regenerative amplifier producing laser pulses with a central wavelength of 1028 nm and at a repetition rate of 2 kHz. The output was divided into two beams for the pump- and probe-pulse generation. One of the beams pumped an optical parametric amplifier (OPA) to generate pump pulses with a wavelength of 643 nm, and the other beam produced probe pulses with a wavelength of 678 nm by using another OPA. The delay time between the pump and probe pulses was controlled by a mechanical delay stage. The circularly polarized pump pulses were obtained using an achromatic quarter-wave plate and a Berek compensator and focused onto the sample by an objective lens [×50, numerical aperture (NA) = 0.42]. The linearly polarized probe pulses counter-propagated with the pump pulses and were loosely focused onto the sample from the glass substrate side by a lens with a focal length of 20 cm. The probe pulses transmitted through the sample were collected by the same objective lens that focused the pump pulses and were then sent to an analyzer composed of an achromatic HWP mounted on a rotation stage and a LP (see Fig. 1A). The transmission axis of the LP was fixed to be perpendicular to the polarization of the incident probe pulses. After passing through the analyzer, the probe pulses were imaged with a CMOS camera. The samples were in a sample holder mounted on a three-axis positioning stage, and the measurements were performed under vacuum conditions at room temperature. The objective lens was designed to compensate the thickness of the optical window of the sample holder.

The polarization rotation angle of the probe pulses transmitted through the sample, θ, was determined from the intensity of the probe pulses detected by the CMOS camera, *I*_d_. The dependence of *I*_d_ on the angle of the achromatic HWP, α, can be written as *I*_d_(α) = *I*_B_ + *I*_T_sin^2^(−2α − θ), where *I*_B_ is the background signal and *I*_T_ is the proportionality constant reflecting the intensity of the incident probe pulses (*38*). To obtain an image of θ, we took images of *I*_d_(α) by changing α from −5 deg to 5 deg with a step of 0.2 deg and fitted the obtained *I*_d_(α) with the relation described above at every pixel position. We measured θ with and without the pump excitation—θ_w_ and θ_wo_—and obtained the pump-induced Faraday rotation angle θ_F_ = θ_w_ − θ_wo_. To prevent the photoluminescence of the sample and the pump pulses from impinging on the CMOS camera, we inserted optical filters in front of the camera. The remaining background signal in the image of *I*_d_(α) with the pump excitation was removed by subtracting the image taken when the probe pulses were blocked from that obtained under irradiation by both the pump and probe pulses. To block the pump and probe pulses separately, we used two optical shutters. The measured images of θ_F_ were smoothed by a median filter with a window size of 5 × 5 pixels to improve the angle resolution (*38*).

### Sample preparation

The (BA)_2_(MA)_3_Pb_4_I_13_ single crystals were synthesized with a slight modification of the procedure reported by Stoumpos *et al.* (*25*). PbI_2_ powder (576 mg, 1.25 mmol) was dissolved in a mixture of 57 weight % (wt %) aqueous HI solution (1.13 ml, 8.6 mmol) and 50 wt % aqueous H_3_PO_2_ solution (125 μl, 1.1 mmol) at 90°C under constant magnetic stirring for about 5 min, which formed a bright yellow solution. Subsequent addition of solid CH_3_NH_3_I (159 mg, 1.5 mmol) to the hot yellow solution initially caused precipitation of a black powder, which was rapidly redissolved under stirring to afford a clear bright yellow solution (solution 1). In a separate vial, *n*-BuNH_2_ (47.5 μl, 0.5 mmol) was neutralized with 57 wt % HI (408 μl, 3.1 mmol) and 50 wt % aqueous H_3_PO_2_ (45.3 ml, 0.41 mmol), resulting in a clear pale yellow solution of *n*-BuNH_3_I (solution 2). Mixing of solutions 1 and 2 initially produced a black precipitate, which was subsequently dissolved by heating the combined solution at 90°C. The stirring was then stopped, and the solution was cooled to 85°C, during which time black rectangular-shaped plates crystallized. The (BA)_2_(MA)_3_Pb_4_I_13_ crystal flakes were mechanically exfoliated and transferred onto a glass substrate in a N_2_-filled glove box.

### Measurement of absorption coefficient spectrum

Absorption coefficient spectra of the exfoliated flakes were obtained by performing reflectance and transmittance measurements. The light from a white light-emitting diode was spatially filtered by a pinhole and focused onto a sample by an objective lens (×50, NA = 0.42). The spot size at full width at half maximum was around 3 μm. For the reflectance measurements, the reflected light was collected by the same objective lens and detected by a spectrometer equipped with a charge-coupled device (CCD) camera. The reflectance spectrum was obtained by taking a ratio of the light intensity reflected from the sample to that reflected from the glass substrate (Matsunami Glass Ind. Ltd.) and multiplying the ratio by the reflectance of the glass substrate estimated from literature values for the refractive index (*53*). For the transmittance measurements, the light transmitted through the sample was collected by another objective lens (×50, NA = 0.42) and detected by the same spectrometer and CCD camera. The transmittance spectrum was obtained by normalizing the light intensity passing through the sample and substrate by that passing through the bare substrate. The absorption coefficient spectrum was determined using the following relations (*54*)

$$R=\frac{L_{-}+M\text{cos}\Phi_{-}}{L_{+}+M\text{cos}\Phi_{+}}$$

$$T=\frac{16(n_{1}^{2}+k_{1}^{2})n_{2}^{2}}{L_{+}+M\text{cos}\Phi_{+}}$$

$$L_{\pm }=a_{\pm }\text{cosh}(4\pi d_{1}k_{1}/\lambda )+b_{\pm }\text{sinh}(4\pi d_{1}k_{1}/\lambda )$$

$$a_{\pm }=(1+n_{1}^{2})(n_{1}^{2}+n_{2}^{2})(n_{2}^{2}+1)\pm 8n_{1}^{2}n_{2}^{2}$$

$$b_{\pm }=4n_{1}(1+n_{1}^{2})n_{2}^{2}\pm 2n_{1}(n_{1}^{2}+n_{2}^{2})(n_{2}^{2}+1)$$

$$M=(1-n_{1}^{2})(n_{1}^{2}-n_{2}^{2})(n_{2}^{2}+1)$$

$$\Phi_{\pm }=\frac{4\pi d_{1}n_{1}}{\lambda }+\varphi_{\pm }$$

$$\varphi_{\pm }={\text{tan}}^{-1}(\frac{m_{\pm }k_{1}}{Mn_{1}})$$

$$m_{\pm }=-4n_{1}(1-n_{1}^{2})n_{2}^{2}\pm 2n_{1}(n_{1}^{2}-n_{2}^{2})(n_{2}^{2}+1)$$

where *R* and *T* are the reflectance and transmittance of the sample, respectively; *n*_1_ and *k*_1_ are the real and imaginary parts of the complex refractive index of the sample, respectively; *d*_1_ is the thickness of the sample; and λ is the wavelength of the light. We obtained the absorption coefficient spectrum α = 2π*k*_1_/λ by using the value of *d*_1_ estimated from atomic force microscopy measurements.

### Relation between γ_s_ and the experimental values

Because of the negligible contributions of the first and second terms on the right-hand side of Eq. 1 within the picosecond time scale, the diffusion equation for the spin-polarized exciton density *n*_+_ − *n*_−_ can be approximated to

$$\frac{\partial (n_{+}-n_{-})}{\partial t}=-\gamma_{s}(n_{+}-n_{-})$$ (3)

In addition, because *n*_+_ + *n*_−_ after the pump excitation can be considered to be constant within the time window of our measurements (see text S5), we can write γ_s_ at the position of *x* = *y* = 0 μm as follows

$$\begin{array}{c} \gamma_{s}(t,x=0,y=0)=\frac{1}{\tau^{\prime }}+2\beta^{\prime }[n_{+}(t,x=0,y=0)+\\ n_{-}(t,x=0,y=0)]=\frac{1}{\tau^{\prime }}+\beta^{\prime }n_{\text{ex}} \end{array}$$ (4)

Here, we used the initial values of *n*_+_(*t* = 0, *x* = 0, *y* = 0) = *n*_ex_/2 and *n*_−_(*t* = 0, *x* = 0, *y* = 0) = 0. From Eqs. 3 and 4, it is found that *n*_+_ − *n*_−_ at *x* = *y* = 0 μm decays with a rate of 1/τ′ + β′*n*_ex_. Therefore, τ′ and β′ correspond to τ_spin_^0^ and β, respectively.

## Supplementary Materials

### This PDF file includes:

Texts S1 to S8 Figs. S1 to S11 References
