# Supplementary material for: Rapidly expanding spin-polarized exciton halo in a two-dimensional halide perovskite at room temperature
Source: Sci Adv. 2022 Jul 29;8(30):eabp8135. doi: 10.1126/sciadv.abp8135 (PMC9337763; doi:10.1126/sciadv.abp8135)
Supplement: Supplementary file 1 — Texts S1 to S8 Figs. S1 to S11 References [file sciadv.abp8135_sm.pdf]

Science Advances
AAAS

Supplementary Materials for

Rapidly expanding spin-polarized exciton halo in a two-dimensional halide perovskite at room temperature

Go Yumoto *et al.*

Corresponding author: Yoshihiko Kanemitsu, kanemitu@scl.kyoto-u.ac.jp

Sci. Adv. **8**, eabp8135 (2022)
DOI: 10.1126/sciadv.abp8135

This PDF file includes:

Texts S1 to S8
Figs. S1 to S11
References

Supplementary Text

S1. Pump-induced Faraday rotation for right- and left-handed circularly polarized pump pulses

In two-dimensional Ruddlesden-Popper lead halide perovskites, the two degenerate band-edge excitons with total exciton angular momentum projection M_{ex} of +1 and −1 determine the optical responses. The excitons with M_{ex} of +1 and −1 can be selectively excited by right-handed (σ^+) or left-handed (σ^-) circularly polarized light, respectively (*13, 17, 18, 20, 21, 23, 35-37, 55*). Therefore it is expected that the pump-induced Faraday rotation angle θ_F does not depend on the pump helicity, except for the sign of θ_F. This is confirmed by comparing θ_F under the pump excitations with the σ^+ and σ^- circular polarizations. Figures S2A and B show the time-resolved images of θ_F for an exciton density per 2D perovskite layer n_{ex} of 1.2×10^{13} cm^{-2} under the pump excitations with the σ^+ and σ^- circular polarizations, respectively. We can see no distinct difference in the spatiotemporal dynamics of θ_F other than its sign.

S2. Dependence of the pump-induced Faraday rotation on the intensity of the probe pulses

To confirm that the probe pulses do not induce nonlinear processes, we measured θ_F for different probe intensities. The radial profiles of θ_F measured by probe pulses with the fluences of 0.68 and 1.37 μJ cm^{-2} are plotted in Fig. S3, where the pump-probe delay time t_{pp} is 0.14 ps and n_{ex} = 1.3×10^{13} cm^{-2}. We found that the observed θ_F does not depend on the probe fluence. This shows that in this range of the probe fluence, the probe pulses do not alter the spatiotemporal dynamics of the pump-induced spin-polarized exciton population. Therefore all measurements were performed with the probe fluence of 1.37 μJ cm^{-2}.

S3. Estimation of the exciton density per 2D perovskite layer

We estimated the exciton density per 2D perovskite layer n_{ex} from the pump photon fluence by taking into account the absorption saturation. The pump photon fluence F can be written by

$$F = \frac{P}{\hbar\omega f_{\mathrm{rep}} \pi \sigma_{x,0} \sigma_{y,0}}\,, \qquad \text{(S1)}$$

where P is the pump power measured at the sample position, $\hbar\omega$ is the pump photon energy, f_{rep} is the laser repetition rate, and $\pi\sigma_{x,0}\sigma_{y,0}$ denotes the pump beam area. Here, $\sigma_{x,0}$ and $\sigma_{y,0}$ are the Gaussian widths defined as $\exp\left(-\frac{x^2}{2\sigma_{x,0}{}^2} - \frac{y^2}{2\sigma_{y,0}{}^2}\right)$. With F and the Lambert-Beer law, we estimated n_{ex} using the following relations:

$$n_{\mathrm{ex}} = F[1 - \exp(-\alpha_{\mathrm{sat}} d)]/(d/d_u)\,, \qquad \text{(S2)}$$

$$\alpha_{\mathrm{sat}} = \frac{\alpha}{1 + P/P_{\mathrm{sat}}}\,, \qquad \text{(S3)}$$

where d is the thickness of the sample, d_u is the total thickness of a 2D perovskite layer ($A_{n-1}Pb_nX_{3n+1}$) and a long organic barrier layer (L_2), α_{sat} and α are respectively the power-dependent and linear absorption coefficients, and P_{sat} is the saturation power (*56*). Here we used the literature value of d_u = 3.22 nm in $(BA)_2(MA)_3Pb_4I_{13}$ crystals (*29*). Equation S3 represents the absorption saturation effect. To estimate P_{sat}, we measured the pump power dependence of the pump-induced change in transmission $\Delta T/T$ right after the pump excitation (see Supplementary Text 5). Figure S4 shows the spatially integrated $\Delta T/T$ as a function of the pump power. Because $\Delta T/T$ is proportional to n_{ex}, the pump power dependence can be fitted using Eqs. S2 and S3, as shown in Fig. S4. From the fit, we obtained P_{sat}.

S4. Reproducibility of the observed halo-like spatial pattern

To clarify that the observed halo-like spatial pattern reflects the intrinsic properties of $(BA)_2(MA)_3Pb_4I_{13}$ crystals, we confirmed that sample degradation does not occur during the measurements. To investigate the effect of the photo-induced degradation, we checked the reproducibility of the radial profiles of θ_F for $n_{ex} = 1.2 \times 10^{13}$ cm^{-2} by performing the measurements first in the descending order of t_{pp} from 4.34 ps to 0.14 ps and then in the ascending order of t_{pp} from 0.14 ps to 4.34 ps (Fig. S5). Figure S5 shows that the radial profiles measured in the descending and ascending orders well agree with each other at all t_{pp}, which shows that no sample degradation occurs during the measurements.

To confirm that the emergence of the halo-like spatial pattern is a common phenomenon in $(BA)_2(MA)_3Pb_4I_{13}$ crystal flakes, we measured the time-resolved images of θ_F in different exfoliated flakes. Figure S6 shows the spatial profiles of θ_F at t_{pp} = 0.14, 2.24, and 4.34 ps for $n_{ex} = 1.3 \times 10^{13}$ cm^{-2} obtained with another flake, the thickness of which was 40 nm. It can be seen that the halo-like spatial pattern appears with increasing t_{pp}. In Fig. S7, we plotted the radial profiles of θ_F at t_{pp} = 4.34 ps for different n_{ex} obtained with the 40-nm-thick flake. By comparing the data shown in Fig. 4A and Fig. S7, we can see that n_{ex} where the halo-like spatial profile appears does not vary between different flakes.

S5. Time-resolved imaging of pump-induced change in transmission

To probe the spatiotemporal dynamics of the total exciton population $N_{+}+N_{-}$, we performed time-resolved imaging of pump-induced change in transmission $\Delta T/T$, which is proportional to $N_{+}+N_{-}$. The time-resolved images of $\Delta T/T$ were obtained using the same experimental setup used for the θ_F measurements but with the analyzer removed. Because the probe photon energy of 1.83 eV used for the θ_F measurements is far from the absorption peak, $\Delta T/T$ measured by the probe pulses is relatively small. Therefore, we used probe pulses with the photon energy of 1.87 eV to measure $\Delta T/T$ with a sufficient signal-to-noise ratio. Figure 1I shows the time-resolved images of $\Delta T/T$ at different t_{pp} for $n_{ex} = 1.3 \times 10^{13}$ cm^{-2} under the σ^+ circularly polarized pump excitation with the photon energy of 1.93 eV. At all t_{pp}, $\Delta T/T$ takes an isotropic Gaussian spatial profile around the center of the excitation spot ($x = y = 0$ μm). This is clearly shown by the fact that the spatial profiles at $y = 0$ μm are well fitted by a Gaussian function (see bottom panels of Fig. 1I). Therefore, we fitted to the spatial profiles of $\Delta T/T$ with a two-dimensional Gaussian function described by $A \times \exp\left(-\frac{x^2}{2\sigma_x{}^2} - \frac{y^2}{2\sigma_y{}^2}\right)$ and derived the temporal dynamics of the peak $\Delta T/T$ and the mean squared width $\sigma^2 = \frac{\sigma_x{}^2+\sigma_y{}^2}{2}$ as shown in Figs. S8A and B. We observed that after the pump excitation $\Delta T/T$ hardly decays and σ^2 slightly increases due to the repulsive exciton-exciton exchange interaction (see Supplementary Text 8).

S6. Estimation of the cross-correlation between the pump and probe pulses

Because the optical Stark shift occurs only during the overlap between the pump and probe pulses (*13*, *37*, *55*, *57*), we measured the time-resolved images of Faraday rotation induced by the optical Stark effect to estimate the cross-correlation between the pump and probe pulses (*58*, *59*). Real excitation of carriers induces a slowly decaying component and obscures the optical Stark effect (*60-62*). Therefore we used $(BA)_2(MA)Pb_2I_7$ crystals (n = 2), which have an exciton resonance energy 0.25 eV larger than the pump photon energy, as shown in the inset of Fig. S9. A detailed description of the sample preparation is given at the end of this section. In Fig. S9, we

plot the t_{pp} dependence of the spatially integrated θ_F under the pump excitations with the σ^+ and σ^- circular polarizations. The laser repetition rate was set to 10 kHz and the pump fluence was 1.3 mJ cm^{-2}. The sign reversal of θ_F depending on the pump polarizations reflects the spin-selective optical Stark effect in lead halide perovskites (*13, 37, 55*). No slowly decaying component can be seen and the data are well fitted with a Gaussian function. From the fitting, we estimated the cross-correlation width between the pump and probe pulses to be 460 fs (full width at half maximum).

Synthesis of $(BA)_2(MA)Pb_2I_7$ (n = 2) single crystals

The $(BA)_2(MA)Pb_2I_7$ single crystals were synthesized with a slight modification of the procedure reported by Stoumpos *et al*. (*25*). PbO powder (2230 mg, 10.0 mmol) was dissolved in a mixture of 57 wt % aqueous HI solution (20.0 ml, 152 mmol) and 50 wt % aqueous H_3PO_2 solution (1.7 ml, 15.5 mmol) at 100 °C under constant magnetic stirring for about 5 min, which formed a bright yellow solution. Subsequent addition of solid CH_3NH_3Cl (335 mg, 5.0 mmol) to the hot yellow solution initially caused the precipitation of a black powder, which was rapidly redissolved under stirring to afford a clear bright yellow solution (solution 1). In a separate vial, *n*-$BuNH_2$ (694 μl, 7.0 mmol) was neutralized with 57 wt % HI (5.0 ml, 38 mmol) in an ice bath, resulting in a clear pale yellow solution of *n*-$BuNH_3I$ (solution 2). Mixing of solutions 1 and 2 initially produced a black precipitate, which was subsequently dissolved by heating the combined solution at 100 °C. The stirring was then stopped, and the solution was left to cool to room temperature, during which time cherry red rectangular-shaped plates crystallized.

S7. Spatial extent of the experimentally observed and calculated spin-polarized exciton population

The two-dimensional diffusion equations including the density-dependent relaxation of exciton spin well describe the observed behavior of the spin-polarized excitons. As clearly shown in Fig. S10, however, the simulated spatial extent of the spin-polarized exciton population is narrower than that observed in the experiments, especially for higher exciton density. Figure S10A shows the experimental and calculated results of the spatial profiles of the spin-polarized exciton population at $y = 0$ μm for $n_{ex} = 6.8 \times 10^{11}$ cm^{-2} (corresponding to the data shown in the bottom panels of Fig. 1F and Fig. 3C in the manuscript), which are normalized by their peak values. A comparable spatial extent is observed in both the experimental and calculated results. On the other hand, the normalized spatial profiles for $n_{ex} = 1.2 \times 10^{13}$ cm^{-2} show that the spatial extent of the experimental result is broader than that of the calculated result (Fig. S10B). This indicates the contribution of ultrafast exciton spin transport, which is more prominent for higher n_{ex}.

S8. Density-dependent ultrafast transport of the total exciton population

As shown in Fig. 4B, the density-dependent ultrafast exciton spin transport is clearly seen from the radial profiles of n_{ex}-normalized θ_F. Along with the exciton spin transport, the density-dependent ultrafast propagation of the total exciton population is observed as shown in Fig. S11. Figure S11 shows the radial profiles of n_{ex}-normalized $\Delta T/T$, $\Delta T/T^{norm}$, at t_{pp} = 1.7 ps for n_{ex} = 4.1 $\times 10^{12}$ and 1.3×10^{13} cm^{-2}. The increase of $\Delta T/T^{norm}$ outside the excitation spot shows that the propagation of the total exciton population occurs within the time window corresponding to τ_{spin}. This agrees with the interpretation that the ultrafast exciton spin transport is driven by the repulsive exciton-exciton exchange interaction.

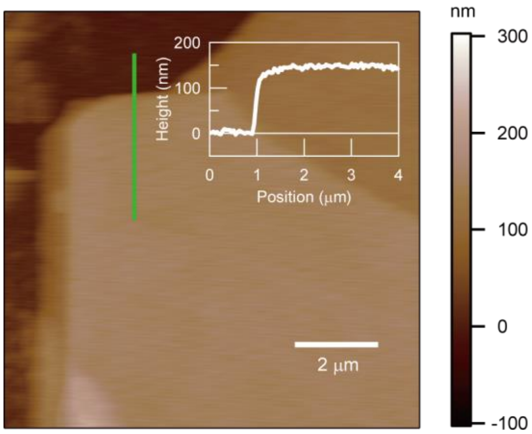

Fig. S1. Atomic force microscopy image. Atomic force microscopy image of the exfoliated flake of the $(BA)_2(MA)_3Pb_4I_{13}$ crystal shown in the inset of Fig. 1D. The inset is a height profile along the green line in the image.

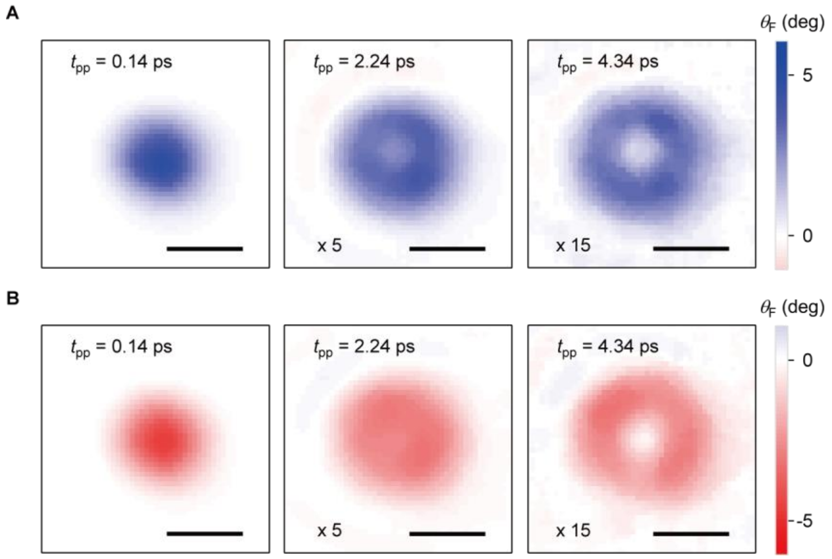

Fig. S2. Time-resolved images of pump-induced Faraday rotation for the right- and left-handed circularly polarized pump pulses. (**A**) Images of pump-induced Faraday rotation angle at pump-probe delay times of 0.14, 2.24, and 4.34 ps for the σ^+ circularly polarized pump pulses. The exciton density is $n_{ex} = 1.2 \times 10^{13}$ cm^{-2}. The middle and right panels are magnified by factors of 5 and 15, respectively. Scale bars are 2 μm. (**B**) Same as (A) but for the σ^- circularly polarized pump pulses.

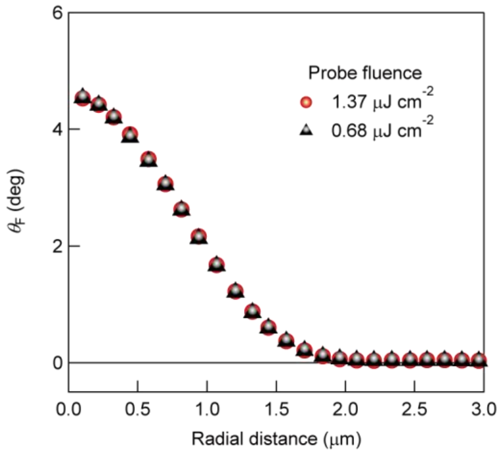

Fig. S3. Spatial profiles of pump-induced Faraday rotation for different probe fluences. Radial profiles of pump-induced Faraday rotation angle for different probe fluences at pump-probe delay time of 0.14 ps for n_{ex} = 1.3×10^{13} cm^{-2}.

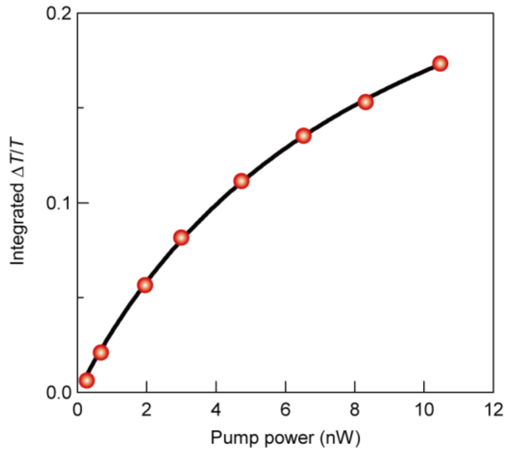

Fig. S4. Pump power dependence of the spatially integrated pump-induced change in transmission. Pump power dependence of the spatially integrated pump-induced change in transmission right after the pump excitation. The black curve is the fit to the data.

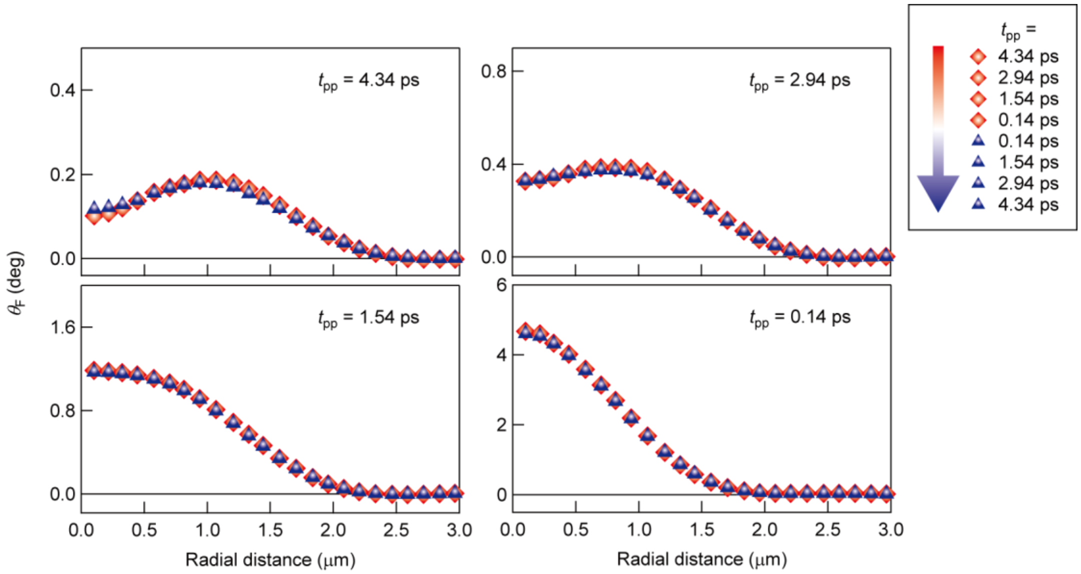

Fig. S5. Radial profiles of pump-induced Faraday rotation measured in the descending and ascending orders of pump-probe delay times. Radial profiles of pump-induced Faraday rotation angle for $n_{ex} = 1.2 \times 10^{13}$ cm^{-2} at pump-probe delay times of 4.34, 2.94, 1.54, and 0.14 ps. The red diamonds and blue triangles respectively correspond to the data measured in descending and ascending orders of the pump-probe delay time. The measurement order is shown in the box.

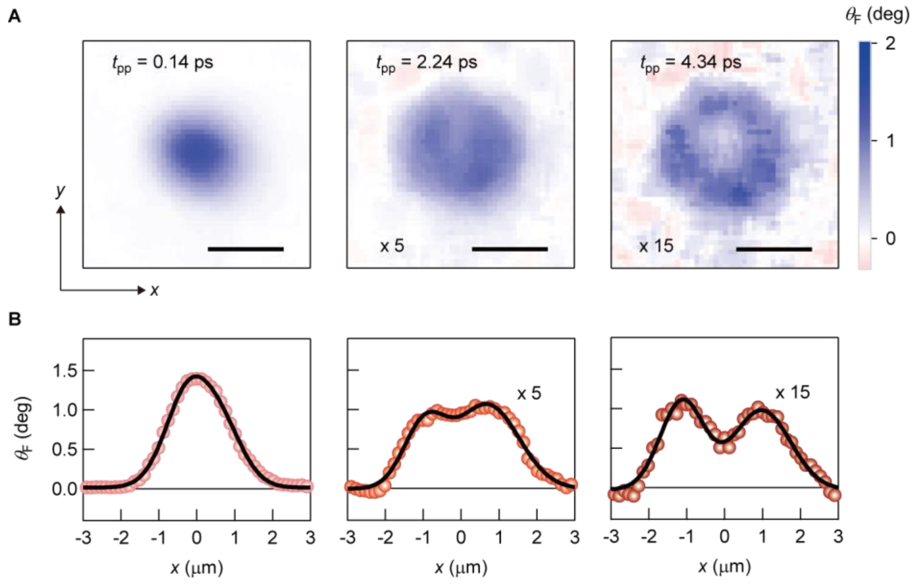

Fig. S6. Spatial profiles of pump-induced Faraday rotation obtained with a different flake. (**A**) Images of pump-induced Faraday rotation angle at pump-probe delay times of 0.14, 2.24, and 4.34 ps for n_{ex} = 1.3 × 10^{13} cm^{-2} obtained with a different flake, whose thickness was 40 nm. The middle and right panels are magnified by factors of 5 and 15, respectively. Scale bars are 2 μm. (**B**) Spatial profiles of pump-induced Faraday rotation angle, which correspond to the horizontal cross sections of the images in (A) passing through the center of the excitation spot (*x* = *y* = 0 μm). The black curves are double-Gaussian fits. The middle and right panels are magnified by factors of 5 and 15, respectively.

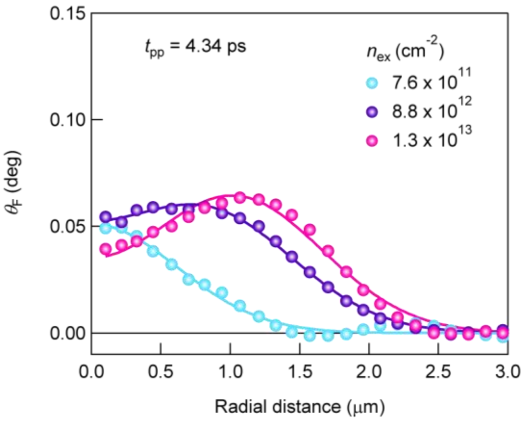

Fig. S7. Exciton density dependence of spatial profiles of pump-induced Faraday rotation obtained with a different flake. Radial profiles of pump-induced Faraday rotation angle for different exciton densities at a pump-probe delay time of 4.34 ps obtained with a different flake, whose thickness was 40 nm. The curves are double-Gaussian fits.

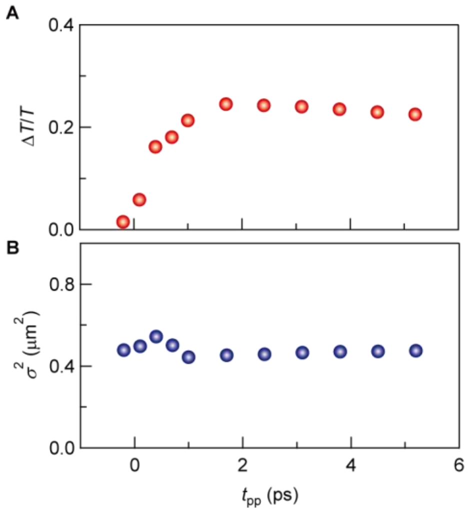

Fig. S8. Spatiotemporal dynamics of pump-induced change in transmission. (**A** and **B**) Estimated peak pump-induced change in transmission (A) and mean squared width (B) as a function of pump-probe delay time for $n_{ex} = 1.3 \times 10^{13}$ cm^{-2}.

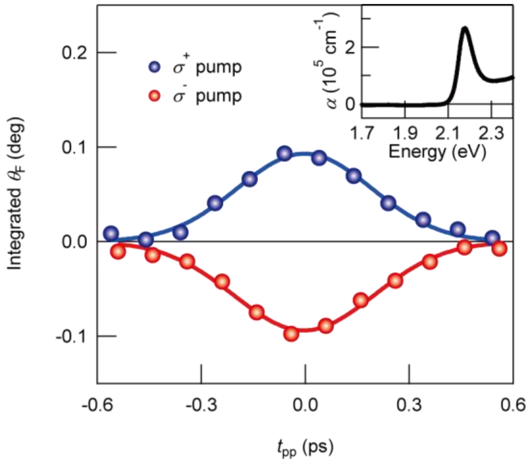

Fig. S9. Temporal dynamics of pump-induced Faraday rotation angle in $(BA)_2(MA)Pb_2I_7$ (*n* = 2) crystals. Spatially integrated pump-induced Faraday rotation angle as a function of pump-probe delay time under the pump excitations with the σ^+ (blue circles) and σ^- (red circles) circular polarizations. The solid curves are Gaussian fits. The inset is an absorption coefficient spectrum of an exfoliated flake of $(BA)_2(MA)Pb_2I_7$ (n = 2) crystals.

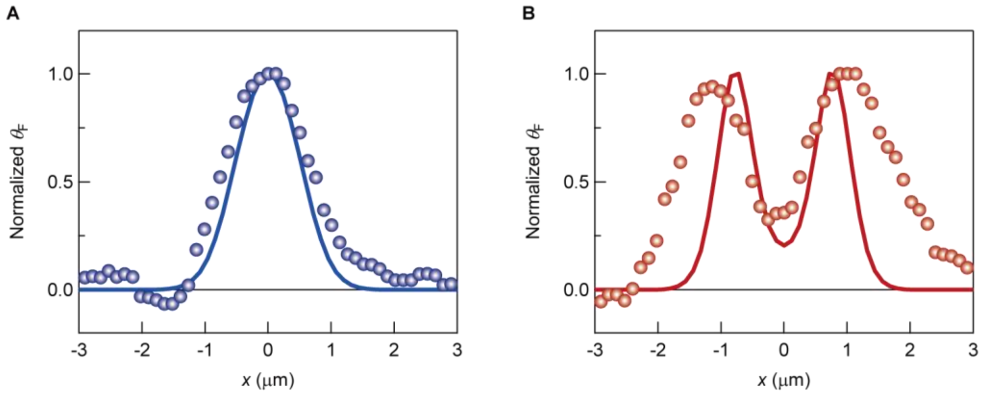

Fig. S10. Experimentally observed and calculated spatial profiles of spin-polarized exciton population. (**A**) Experimentally obtained (solid circles) and calculated (solid curve) spatial profiles of pump-induced Faraday rotation angle at y = 0 µm for $n_{ex} = 6.8 \times 10^{11}$ cm^{-2}. These data correspond to the spatial profiles shown in the bottom panels of Fig. 1F and Fig. 3C in the manuscript. Both profiles are normalized by their peak values. (**B**) Same as (A) but for $n_{ex} = 1.2 \times 10^{13}$ cm^{-2}. The profiles correspond to the data shown in the bottom panels of Fig. 1H and Fig. 3D in the manuscript.

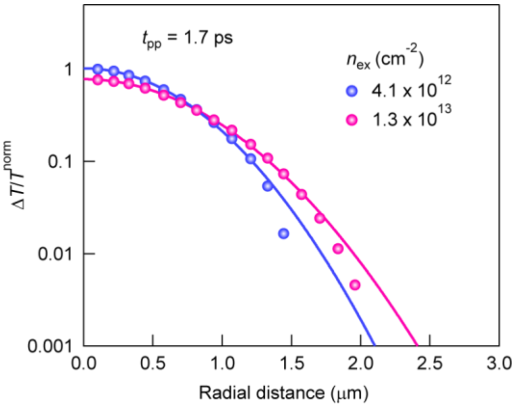

Fig. S11. Exciton density dependence of spatial profiles of pump-induced change in transmission. Radial profiles of pump-induced change in transmission at a pump-probe delay time of 1.7 ps for $n_{ex} = 4.1 \times 10^{12}$ and 1.3×10^{13} cm^{-2}. The radial profiles are normalized by each exciton density and further multiplied by a factor that makes the peak value for the lower exciton density equal to 1. The normalized pump-induced change in transmission is shown in log scale. The curves are Gaussian fits.

REFERENCES AND NOTES

1. M. J. Stevens, A. L. Smirl, R. D. R. Bhat, A. Najmaie, J. E. Sipe, H. M. van Driel, Quantum interference control of ballistic pure spin currents in semiconductors. *Phys. Rev. Lett.* **90**, 136603 (2003).

2. Y. K. Kato, R. C. Myers, A. C. Gossard, D. D. Awschalom, Observation of the spin Hall effect in semiconductors. *Science* **306**, 1910–1913 (2004).

3. J. R. Leonard, Y. Y. Kuznetsova, S. Yang, L. V. Butov, T. Ostatnický, A. Kavokin, A. C. Gossard, Spin transport of excitons. *Nano Lett.* **9**, 4204–4208 (2009).

4. M. P. Walser, C. Reichl, W. Wegscheider, G. Salis, Direct mapping of the formation of a persistent spin helix. *Nat. Phys.* **8**, 757–762 (2012).

5. P. Rivera, K. L. Seyler, H. Yu, J. R. Schaibley, J. Yan, D. G. Mandrus, W. Yao, X. Xu, Valley-polarized exciton dynamics in a 2D semiconductor heterostructure. *Science* **351**, 688–691 (2016).

6. M. Onga, Y. Zhang, T. Ideue, Y. Iwasa, Exciton Hall effect in monolayer MoS_2. *Nat. Mater.* **16**, 1193–1197 (2017).

7. C. Jin, J. Kim, M. I. B. Utama, E. C. Regan, H. Kleemann, H. Cai, Y. Shen, M. J. Shinner, A. Sengupta, K. Watanabe, T. Taniguchi, S. Tongay, A. Zettl, F. Wang, Imaging of pure spin-valley diffusion current in WS_2-WSe_2 heterostructures. *Science* **360**, 893–896 (2018).

8. D. Unuchek, A. Ciarrocchi, A. Avsar, Z. Sun, K. Watanabe, T. Taniguchi, A. Kis, Valley-polarized exciton currents in a van der Waals heterostructure. *Nat. Nanotechnol.* **14**, 1104–1109 (2019).

9. K. F. Mak, D. Xiao, J. Shan, Light–valley interactions in 2D semiconductors. *Nat. Photonics* **12**, 451–460 (2018).

10. C. Jin, E. Y. Ma, O. Karni, E. C. Regan, F. Wang, T. F. Heinz, Ultrafast dynamics in van der Waals heterostructures. *Nat. Nanotechnol.* **13**, 994–1003 (2018).

11. L. Sun, C.-Y. Wang, A. Krasnok, J. Choi, J. Shi, J. S. Gomez-Diaz, A. Zepeda, S. Gwo, C.-K. Shih, A. Alù, X. Li, Separation of valley excitons in a MoS_2 monolayer using a subwavelength asymmetric groove array. *Nat. Photonics* **13**, 180–184 (2019).

12. O. Kyriienko, E. B. Magnusson, I. A. Shelykh, Spin dynamics of cold exciton condensates. *Phys. Rev. B* **86**, 115324 (2012).

13. D. Giovanni, W. K. Chong, H. A. Dewi, K. Thirumal, I. Neogi, R. Ramesh, S. Mhaisalkar, N. Mathews, T. C. Sum, Tunable room-temperature spin-selective optical Stark effect in solution-processed layered halide perovskites. *Sci. Adv.* **2**, e1600477 (2016).

14. H. Tsai, W. Nie, J.-C. Blancon, C. C. Stoumpos, R. Asadpour, B. Harutyunyan, A. J. Neukirch, R. Verduzco, J. J. Crochet, S. Tretiak, L. Pedesseau, J. Even, M. A. Alam, G. Gupta, J. Lou, P. M. Ajayan, M. J. Bedzyk, M. G. Kanatzidis, A. D. Mohite, High-efficiency two-dimensional Ruddlesden–Popper perovskite solar cells. *Nature* **536**, 312–316 (2016).

15. M. Yuan, L. N. Quan, R. Comin, G. Walters, R. Sabatini, O. Voznyy, S. Hoogland, Y. Zhao, E. M. Beauregard, P. Kanjanaboos, Z. Lu, D. H. Kim, E. H. Sargent, Perovskite energy funnels for efficient light-emitting diodes. *Nat. Nanotechnol.* **11**, 872–877 (2016).

16. Y. Zhai, S. Baniya, C. Zhang, J. Li, P. Haney, C.-X. Sheng, E. Ehrenfreund, Z. V. Vardeny, Giant Rashba splitting in 2D organic-inorganic halide perovskites measured by transient spectroscopies. *Sci. Adv.* **3**, e1700704 (2017).

17. D. Giovanni, W. K. Chong, Y. Y. F. Liu, H. A. Dewi, T. Yin, Y. Lekina, Z. X. Shen, N. Mathews, C. K. Gan, T. C. Sum, Coherent spin and quasiparticle dynamics in solution-processed layered 2D lead halide perovskites. *Adv. Sci.* **5**, 1800664 (2018).

18. X. Chen, H. Lu, Z. Li, Y. Zhai, P. F. Ndione, J. J. Berry, K. Zhu, Y. Yang, M. C. Beard, Impact of layer thickness on the charge carrier and spin coherence lifetime in two-dimensional layered perovskite single crystals. *ACS Energy Lett.* **3**, 2273–2279 (2018).

19. D. Giovanni, J. W. M. Lim, Z. Yuan, S. S. Lim, M. Righetto, J. Qing, Q. Zhang, H. A. Dewi, F. Gao, S. G. Mhaisalkar, N. Mathews, T. C. Sum, Ultrafast long-range spin-funneling in solution-processed Ruddlesden–Popper halide perovskites. *Nat. Commun.* **10**, 3456 (2019).

20. S. B. Todd, D. B. Riley, A. Binai-Motlagh, C. Clegg, A. Ramachandran, S. A. March, J. M. Hoffman, I. G. Hill, C. C. Stoumpos, M. G. Kanatzidis, Z.-G. Yu, K. C. Hall, Detection of Rashba spin splitting in 2D organic-inorganic perovskite via precessional carrier spin relaxation. *APL Mater.* **7**, 081116 (2019).

21. S. A. Bourelle, R. Shivanna, F. V. A. Camargo, S. Ghosh, A. J. Gillett, S. P. Senanayak, S. Feldmann, L. Eyre, A. Ashoka, T. W. J. van de Goor, H. Abolins, T. Winkler, G. Cerullo, R. H. Friend, F. Deschler, How exciton interactions control spin-depolarization in layered hybrid perovskites. *Nano Lett.* **20**, 5678–5685 (2020).

22. X. Liu, A. Chanana, U. Huynh, F. Xue, P. Haney, S. Blair, X. Jiang, Z. V. Vardeny, Circular photogalvanic spectroscopy of Rashba splitting in 2D hybrid organic–inorganic perovskite multiple quantum wells. *Nat. Commun.* **11**, 323 (2020).

23. X. Chen, H. Lu, K. Wang, Y. Zhai, V. Lunin, P. C. Sercel, M. C. Beard, Tuning spin-polarized lifetime in two-dimensional metal–halide perovskite through exciton binding energy. *J. Am. Chem. Soc.* **143**, 19438–19445 (2021).

24. K. Cong, E. Vetter, L. Yan, Y. Li, Q. Zhang, Y. Xiong, H. Qu, R. D. Schaller, A. Hoffmann, A. F. Kemper, Y. Yao, J. Wang, W. You, H. Wen, W. Zhang, D. Sun, Coherent control of asymmetric spintronic terahertz emission from two-dimensional hybrid metal halides. *Nat. Commun.* **12**, 5744 (2021).

25. C. C. Stoumpos, D. H. Cao, D. J. Clark, J. Young, J. M. Rondinelli, J. I. Jang, J. T. Hupp, M. G. Kanatzidis, Ruddlesden–Popper hybrid lead iodide perovskite 2D homologous semiconductors. *Chem. Mater.* **28**, 2852–2867 (2016).

26. T. Ishihara, J. Takahashi, T. Goto, Exciton state in two-dimensional perovskite semiconductor $(C_{10}H_{21}NH_3)_2PbI_4$. *Solid State Commun.* **69**, 933–936 (1989).

27. K. Tanaka, T. Takahashi, T. Kondo, T. Umebayashi, K. Asai, K. Ema, Image charge effect on two-dimensional excitons in an inorganic-organic quantum-well crystal. *Phys. Rev. B* **71**, 045312 (2005).

28. O. Yaffe, A. Chernikov, Z. M. Norman, Y. Zhong, A. Velauthapillai, A. van der Zande, J. S. Owen, T. F. Heinz, Excitons in ultrathin organic-inorganic perovskite crystals. *Phys. Rev. B* **92**, 045414 (2015).

29. J. C. Blancon, A. V. Stier, H. Tsai, W. Nie, C. C. Stoumpos, B. Traoré, L. Pedesseau, M. Kepenekian, F. Katsutani, G. T. Noe, J. Kono, S. Tretiak, S. A. Crooker, C. Katan, M. G. Kanatzidis, J. J. Crochet, J. Even, A. D. Mohite, Scaling law for excitons in 2D perovskite quantum wells. *Nat. Commun.* **9**, 2254 (2018).

30. X. Wu, M. T. Trinh, X. Y. Zhu, Excitonic many-body interactions in two-dimensional lead iodide perovskite quantum wells. *J. Phys. Chem. C* **119**, 14714–14721 (2015).

31. G. Delport, G. Chehade, F. Lédée, H. Diab, C. Milesi-Brault, G. Trippé-Allard, J. Even, J.-S. Lauret, E. Deleporte, D. Garrot, Exciton–exciton annihilation in two-dimensional halide perovskites at room temperature. *J. Phys. Chem. Lett.* **10**, 5153–5159 (2019).

32. S. Deng, E. Shi, L. Yuan, L. Jin, L. Dou, L. Huang, Long-range exciton transport and slow annihilation in two-dimensional hybrid perovskites. *Nat. Commun.* **11**, 664 (2020).

33. J. Even, L. Pedesseau, M. A. Dupertuis, J. M. Jancu, C. Katan, Electronic model for self-assembled hybrid organic/perovskite semiconductors: Reverse band edge electronic states ordering and spin-orbit coupling. *Phys. Rev. B* **86**, 205301 (2012).

34. J. Even, L. Pedesseau, J.-M. Jancu, C. Katan, Importance of spin–orbit coupling in hybrid organic/inorganic perovskites for photovoltaic applications. *J. Phys. Chem. Lett.* **4**, 2999–3005 (2013).

35. D. Giovanni, H. Ma, J. Chua, M. Grätzel, R. Ramesh, S. Mhaisalkar, N. Mathews, T. C. Sum, Highly spin-polarized carrier dynamics and ultralarge photoinduced magnetization in $CH_3NH_3PbI_3$ perovskite thin films. *Nano Lett.* **15**, 1553–1558 (2015).

36. P. Odenthal, W. Talmadge, N. Gundlach, R. Wang, C. Zhang, D. Sun, Z.-G. Yu, Z. Valy Vardeny, Y. S. Li, Spin-polarized exciton quantum beating in hybrid organic–inorganic perovskites. *Nat. Phys.* **13**, 894–899 (2017).

37. G. Yumoto, H. Hirori, F. Sekiguchi, R. Sato, M. Saruyama, T. Teranishi, Y. Kanemitsu, Strong spin-orbit coupling inducing Autler-Townes effect in lead halide perovskite nanocrystals. *Nat. Commun.* **12**, 3026 (2021).

38. Y. Hashimoto, A. R. Khorsand, M. Savoini, B. Koene, D. Bossini, A. Tsukamoto, A. Itoh, Y. Ohtsuka, K. Aoshima, A. V. Kimel, A. Kirilyuk, T. Rasing, Ultrafast time-resolved magneto-optical imaging of all-optical switching in GdFeCo with femtosecond time-resolution and a μm spatial-resolution. *Rev. Sci. Instrum.* **85**, 063702 (2014).

39. J. Zhang, S. Langner, J. Wu, C. Kupfer, L. Lüer, W. Meng, B. Zhao, C. Liu, M. Daum, A. Osvet, N. Li, M. Halik, T. Stubhan, Y. Zhao, J. A. Hauch, C. J. Brabec, Intercalating-organic-cation-induced stability bowing in quasi-2D metal-halide perovskites. *ACS Energy Lett.* **7**, 70–77 (2021).

40. N. N. Udalova, S. A. Fateev, E. M. Nemygina, A. Zanetta, G. Grancini, E. A. Goodilin, A. B. Tarasov, Nonmonotonic photostability of $BA_2MA_{n-1}Pb_nI_{3n+1}$ homologous layered perovskites. *ACS Appl. Mater. Interfaces* **14**, 961–970 (2022).

41. K. Leng, I. Abdelwahab, I. Verzhbitskiy, M. Telychko, L. Chu, W. Fu, X. Chi, N. Guo, Z. Chen, Z. Chen, C. Zhang, Q.-H. Xu, J. Lu, M. Chhowalla, G. Eda, K. P. Loh, Molecularly thin two-dimensional hybrid perovskites with tunable optoelectronic properties due to reversible surface relaxation. *Nat. Mater.* **17**, 908–914 (2018).

42. W. Tao, Q. Zhou, H. Zhu, Dynamic polaronic screening for anomalous exciton spin relaxation in two-dimensional lead halide perovskites. *Sci. Adv.* **6**, eabb7132 (2020).

43. R. A. Kaindl, D. Hägele, M. A. Carnahan, D. S. Chemla, Transient terahertz spectroscopy of excitons and unbound carriers in quasi-two-dimensional electron-hole gases. *Phys. Rev. B* **79**, 045320 (2009).

44. M. Kulig, J. Zipfel, P. Nagler, S. Blanter, C. Schüller, T. Korn, N. Paradiso, M. M. Glazov, A. Chernikov, Exciton diffusion and halo effects in monolayer semiconductors. *Phys. Rev. Lett.* **120**, 207401 (2018).

45. M. M. Glazov, Phonon wind and drag of excitons in monolayer semiconductors. *Phys. Rev. B* **100**, 045426 (2019).

46. R. Perea-Causín, S. Brem, R. Rosati, R. Jago, M. Kulig, J. D. Ziegler, J. Zipfel, A. Chernikov, E. Malic, Exciton propagation and halo formation in two-dimensional materials. *Nano Lett.* **19**, 7317–7323 (2019).

47. T. Amand, D. Robart, X. Marie, M. Brousseau, P. Le Jeune, J. Barrau, Spin relaxation in polarized interacting exciton gas in quantum wells. *Phys. Rev. B* **55**, 9880–9896 (1997).

48. F. Mahmood, Z. Alpichshev, Y.-H. Lee, J. Kong, N. Gedik, Observation of exciton–exciton interaction mediated valley depolarization in monolayer $MoSe_2$. *Nano Lett.* **18**, 223–228 (2018).

49. Z. Guo, Y. Wan, M. Yang, J. Snaider, K. Zhu, L. Huang, Long-range hot-carrier transport in hybrid perovskites visualized by ultrafast microscopy. *Science* **356**, 59–62 (2017).

50. J. Sung, C. Schnedermann, L. Ni, A. Sadhanala, R. Y. S. Chen, C. Cho, L. Priest, J. M. Lim, H.-K. Kim, B. Monserrat, P. Kukura, A. Rao, Long-range ballistic propagation of carriers in methylammonium lead iodide perovskite thin films. *Nat. Phys.* **16**, 171–176 (2020).

51. J. Sung, S. Macpherson, A. Rao, Enhanced ballistic transport of charge carriers in alloyed and K-passivated alloyed perovskite thin films. *J. Phys. Chem. Lett.* **11**, 5402–5406 (2020).

52. A. L. Ivanov, Quantum diffusion of dipole-oriented indirect excitons in coupled quantum wells. *Europhys. Lett.* **59**, 586–591 (2002).

53. Z. Zhang, D.-F. Lu, Z.-M. Qi, Application of porous TiO_2 thin films as wavelength-interrogated waveguide resonance sensors for bio/chemical detection. *J. Phys. Chem. C* **116**, 3342–3348 (2012).

54. H. P. Pasanen, P. Vivo, L. Canil, A. Abate, N. Tkachenko, Refractive index change dominates the transient absorption response of metal halide perovskite thin films in the near infrared. *Phys. Chem. Chem. Phys.* **21**, 14663–14670 (2019).

55. Y. Yang, M. Yang, K. Zhu, J. C. Johnson, J. J. Berry, J. van de Lagemaat, M. C. Beard, Large polarization-dependent exciton optical Stark effect in lead iodide perovskites. *Nat. Commun.* **7**, 12613 (2016).

56. R. W. Boyd, *Nonlinear Optics* (Academic Press, ed. 3, 2008).

57. E. J. Sie, J. W. McIver, Y.-H. Lee, L. Fu, J. Kong, N. Gedik, Valley-selective optical Stark effect in monolayer WS_2. *Nat. Mater.* **14**, 290–294 (2015).

58. M. Combescot, O. Betbeder-Matibet, Faraday rotation in photoexcited semiconductors: A composite-exciton many-body effect. *Phys. Rev. B* **74**, 125316 (2006).

59. T. LaMountain, H. Bergeron, I. Balla, T. K. Stanev, M. C. Hersam, N. P. Stern, Valley-selective optical Stark effect probed by Kerr rotation. *Phys. Rev. B* **97**, 045307 (2018).

60. N. Peyghambarian, S. W. Koch, M. Lindberg, B. Fluegel, M. Joffre, Dynamic Stark effect of exciton and continuum states in CdS. *Phys. Rev. Lett.* **62**, 1185–1188 (1989).

61. E. J. Sie, C. H. Lui, Y.-H. Lee, J. Kong, N. Gedik, Observation of intervalley biexcitonic optical Stark effect in monolayer WS_2. *Nano Lett.* **16**, 7421–7426 (2016).

62. S. Sim, D. Lee, J. Lee, H. Bae, M. Noh, S. Cha, M.-H. Jo, K. Lee, H. Choi, Light polarization-controlled conversion of ultrafast coherent–incoherent exciton dynamics in few-layer ReS_2. *Nano Lett.* **19**, 7464–7469 (2019).
